# In vitro safety assessment of nanosilver with improved cell culture systems

**DOI:** 10.1186/1753-6561-7-S6-P10

**Published:** 2013-12-04

**Authors:** Alina Martirosyan, Madeleine Polet, Yves-Jacques Schneider

**Affiliations:** 1 Laboratory of Cellular, Nutritional and Toxicological Biochemistry, Institute of Life Sciences & UCLouvain, Croix du Sud, L7.07.03, Louvain-la-Neuve, B1348, Belgium

## Background

Silver nanoparticles (Ag-NPs) become increasingly prevailing in consumer products as antibacterial agents [1] and their potential threat on human health makes the risk assessment of utmost importance. In order to elucidate the complex interactions of Ag-NPs upon digestion in the gastrointestinal tract, an improved *in vitro *cell culture system was used. The model contained, beside the enterocytes, specialized microfold (M) cells, able to increase the absorption of micro- and nanoparticles [2,3].

In the current study, different aspects of the toxicity of Ag-NPs on the cell of intestinal epithelium were studied, *i.e*. cytotoxicity, inflammatory response and barrier integrity of the epithelial monolayer.

## Materials and methods

The cytotoxic effect of AgNPs < 20 nm (10-90 μg/ml, Mercator GmbH, DE) was assessed by MTT assay on Caco-2 cells (clone 1, from Dr. M. Rescigno, University of Milano-Bicocca, IT). The co-culture model was received by co-culturing Caco-2 cells with RajiB cells (ATCC, Manassas, VA) in Transwell permeable supports (Corning Inc., NY) [1,2]. The inflammatory mediators chemokine IL-8 and nitric oxide (NO) levels were analysed in both apical (AP) and basolateral (BL) compartments by ELISA (BD Biosciences Pharmingen, San Diego, CA) and by Nitrate/Nitrite Colorimetric Assay Kit (Cayman Chemical Company, Ann Arbor, MI), respectively, according to the manufacturer's instructions.

The expression levels of the IL-8 and iNOS (inducible Nitric Oxide Synthase) genes were evaluated by quantitative real-time PCR (qRT-PCR), where the primers used were: for IL-8 CTGGCCGTGGCTCTCTTG (sense) and GGGTGGAAAGGTTTGGAGTATG (antisense) and for iNOS - TGTGCCACCTCCAGTCCAGT (sense) CTTATGGTGAAGTGTGTCTTGGAA (antisense). Levels of individual transcripts were normalized to those of glyceraldehyde-3-phosphate dehydrogenase (GAPDH). Relative quantification (RQ) values - fold change of the target gene expression compared to the untreated sample, were calculated by 2^-ΔΔCt ^method [4].

The barrier integrity of the cell monolayers of mono- and co-cultures under the influence of AgNPs was evaluated on 21 days fully differentiated cultures in bicameral inserts by measuring the transepithelial electrical resistance and the passage of Lucifer Yellow.

The immunofluorescence staining of two tight junctions (TJs) proteins, *i.e*. occludin and ZO-1 was realized by mouse anti-occludin/anti-ZO-1 as primary and Alexa Fluor 488 goat anti-mouse as secondary antibodies (Invitrogen). Images were collected by Zeiss LSM 710 confocal microscope.

## Results

**Figure 1:**
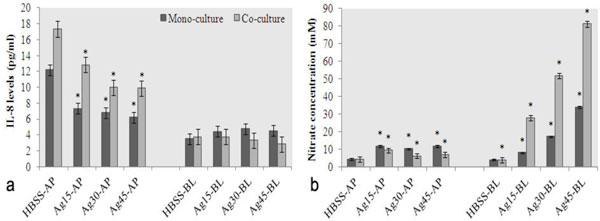
**IL-8 (a) and NO (b) levels in mono- and co-cultures in AP and BL compartments upon exposure to Ag-NPs (45 μg/ml)**. *samples significantly different from the corresponding control. Means of 3 independent experiments ± SD are given, P < 0.001.

Ag-NPs displayed a dose-dependent cytotoxic effect on Caco-2 cells starting from 30 μg/ml. The pro-inflammatory chemokine IL-8 levels were reduced under the influence of Ag-NPs (Figure 1a) in AP compartments in both mono- and co-cultures. In contrast, practically no changes in IL-8 levels were observed in the BL compartments. The ELISA analysis data were confirmed by qRT-PCR analysis, where the expression levels of the IL-8 gene showed a tendency to decrease in both mono- (fold change ≈ 0.86) and co-cultures (fold change ≈ 0.7) under the influence of Ag-NPs.

NO content was increased in both AP and BL compartments in both mono- and co-cultures (Figure 1b), although more marked in the latter case. In BL compartments, the NO levels increase was dependent on the Ag-NPs concentration. In contrast to IL-8, there were practically no changes observed in the iNOS gene expression levels in Caco-2 cells, indicating that Ag-NP-induced NO generation increase is likely independent of the iNOS gene expression.

Immunostaining with confocal microscopy analysis of two TJs proteins, *i.e*. occludin and ZO-1, revealed that, in Ag-NP-treated cells, the continuity of both occludin and ZO-1 was disrupted as compared to control and the aggregation of both proteins was observed. The Ag-NP-induced dashed and degraded distributions of occludin and ZO-1 suggest the opening of TJs (not illustrated). The opening of junctions was further confirmed by decreased TEER values and increased LY passage rates in Ag-NP-treated samples. These effects were less obvious in co-cultures, a more accurate model to reflect *in vivo *conditions, suggesting that the presence of M-cells seemingly decreases the toxicity of AgNPs.

## Conclusions

These results suggest that Ag-NPs: (i) are cytotoxic for intestinal epithelial cells; (ii) possess anti-inflammatory properties; and (iii) mediate the intestinal barrier function disruption. Differences in response to Ag-NPs were observed in mono- and co-cultures, where the NPs affected less obviously the IL-8 levels and barrier function in co-cultures, while, in contrast, led to more marked increase of NO concentration in comparison with mono-cultures. These differences demonstrate the advisability of application of more complex *in vitro *models and further need of improvement of the model by addition of e.g. mucus producing cells and/or dendritic cells that would provide a tool to achieve even more reliable and predictive correlations between *in vitro *studies and *in vivo *outcomes.
